# Muscle Protein Hydrolysates and Amino Acid Composition in Fish

**DOI:** 10.3390/md19070377

**Published:** 2021-06-29

**Authors:** Bomi Ryu, Kyung-Hoon Shin, Se-Kwon Kim

**Affiliations:** 1 Department of Marine Life Science, Jeju National University, Jeju 63243, Korea; 2 Department of Marine Science and Convergence Engineering, Hanyang University, Erica, 55 Hanyangdae-ro, Ansan-si 11558, Gyeonggi-do, Korea; shinkh@hanyang.ac.kr

**Keywords:** fish muscle, amino acid, protein hydrolysate, enzyme, bioactivity, human health

## Abstract

Fish muscle, which accounts for 15%–25% of the total protein in fish, is a desirable protein source. Their hydrolysate is in high demand nutritionally as a functional food and thus has high potential added value. The hydrolysate contains physiologically active amino acids and various essential nutrients, the contents of which depend on the source of protein, protease, hydrolysis method, hydrolysis conditions, and degree of hydrolysis. Therefore, it can be utilized for various industrial applications including use in nutraceuticals and pharmaceuticals to help improve the health of humans. This review discusses muscle protein hydrolysates generated from the muscles of various fish species, as well as their amino acid composition, and highlights their functional properties and bioactivity. In addition, the role of the amino acid profile in regulating the biological and physiological activities, nutrition, and bitter taste of hydrolysates is discussed.

## 1. Introduction

The use of fish and seafood products, including aquatic plants, has increased by ~8% over the last 30 years, with high growth rates of global livestock production [1]. From 1986 to 2016, the annual consumption of fishery products increased from 71.8 million tons to 148.2 million tons, followed by 156.4 million tons in 2018, due to a continuous supply of fish for human consumption through the development of aquaculture and the allowance of captive fisheries [2]. Between 1961 and 2018, the average annual rise in global food fish consumption of 3.1% had outpaced the population growth (1.6%) and exceeded the consumption escalation of all terrestrial animal products combined (such as beef, poultry, and milk), which increased by 2.1% per annum [2,3]. The total initial sales of fishery products in 2016 was USD 362 billion, which increased to USD 401 billion in 2018. In particular, the global fishery market in 2030 is expected to increase by 18% compared to 2018 due to the continuous increase in fishery production, income increase due to urbanization, and changes in eating habits [2].

Various clinical studies have identified the health-promoting effects of consuming fish proteins. Mozaffarian et al. (2005, [4]) found that 4775 adults aged >65 years who consumed tuna or other grilled fish had a lower risk of ischemic stroke. Zheng et al. (2012, [5]) also reported a lower incidence of ischemic heart disease in 3910 adults aged >65 years who consumed tuna or other grilled fish. This suggests that fish consumption reduces the risk of cardiovascular diseases among elderly persons. A population-based prospective cohort study by Matsuoka et al. (2017) found that among Japanese persons aged 40–59 years in 1990 and 40–69 years in 1993, fish consumption equivalent to 111 mg of eicosapentaenoic acid (EPA) or 123 mg of docosahexaenoic acid (DHA) per day was associated with a reduced risk of major depressive disorder [6]. Rylander reported that consuming 75–100 g/day of lean fish conferred benefits on 33,740 Norwegian women aged 40–55 years with type 2 diabetes mellitus. They noted that further investigation is needed to determine whether lean fish itself protects against type 2 diabetes, or whether lean fish consumption affects consumer lifestyles [7].

Fish muscle, which contains 15%–25% of total protein in fish, can be divided into myofibrillar (50%–60%), sarcoplasmic (30%), and stromal (10%–20%) proteins [8]. The muscle proteins have abundant essential amino acids, such as lysine, tryptophan, histidine, phenylalanine, leucine, isoleucine, threonine, and methionine–cystine, accounting for 9.3%, 1.0%, 2.1%, 4.2%, 8.9%, 6.4%, 4.9%, and 4.2% of the total protein content, respectively [9]. Because of the high demand for better pharmaceutical and nutritional products, interest in high-quality nutritional components and in the various bioactivities of the hydrolysate of fish muscle protein with high sensitivity to proteolytic breakdown is increasing [10]. Therefore, efforts have been directed toward full absorption of essential nutrients and amino acids present in fish by hydrolyzing fish proteins. In addition, in terms of their natural availability, relatively low-cost extraction methods, and ability to exert beneficial effects on human health, fish muscle protein hydrolysates and their amino acids are desirable functional ingredients [11]. This review examines the status of recent research findings on fish protein hydrolysates and their proximal components, amino acid composition, and functional and biological properties. 

## 2. Characteristics of Fish Muscle Protein

Human food is produced from protein that comprises ~60% of the body weight of fish. Skeletal muscle proteins in fish facilitate muscle movement by contraction. The heterogeneous fibrous populations of cells in fish proteins differ in terms of molecular, structural, contractile, and metabolic functions and influence the growth rate and properties of muscle, such as the onset of firmness, pH decline, color, and cohesiveness, and ultimately the functional properties of meat [9,12,13]. The firmness of muscle varies with cellularity, including muscle fiber density and diameter; therefore, the flesh of fish is softer than that of terrestrial animals as constant energy is not needed to support their skeletons in water [14]. 

**Figure 1:**
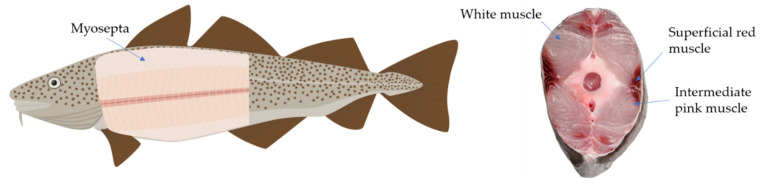
Whole fish and sectional slide with myosepta, major white muscle, a superficial red muscle, and an intermediate pink muscle.

In contrast to terrestrial animals, fish have shorter muscle fibers and less connective tissue, and these muscles are segmented into myotomes by fine connective tissue layers called myocommata or myosepta, which lie primarily in thin sheets that separate muscle fibers into orderly layers [15,16]. In addition, muscle fiber in fish is categorized into three main types of muscle, namely a major white muscle, a superficial red muscle, and an intermediate pink muscle; the axial muscle consists mainly of fast white fibers, covered by a thin layer of slow-red muscle fibers at the periphery, with a layer of pink intermediate muscle fibers in between them (Figure 1, [17]). Salmonids lack the intermediate fiber type. Muscle fibers become shorter towards the tail end of the filet, and connective tissue forms a supporting network throughout the whole fish muscle. Although the connective tissue content is low but more evenly distributed in muscles of fish than in terrestrial animals, firmness increases along the anterior–posterior axis of the filet. 

Muscle proteins from fish are easily digestible and rich in more essential amino acids than most terrestrial meat proteins [3]. Fish muscle protein contains a well-balanced amino acid composition; specifically, Lys accounts for 8.8%, Trp 1.0%, His 2.0%, Phe 3.9%, Leu 8.4%, Ile 6.0%, Thr 4.6%, Met-Cys 4.0%, and Val 6.0% [18]. The consumption of fish muscle protein is associated with health benefits, particularly anti-inflammatory, antioxidant, and angiotensin-converting enzyme inhibitory activities and antimicrobial effects [10,19,20,21,22,23]. Hydrolysates of fish muscle proteins with good nutritional composition, amino acid profiles, and biological activities are easily absorbed in the gastrointestinal tract, and their bioavailability and stability due to decreased fragments have attracted attention in terms of producing valuable food ingredients. Current knowledge of muscle protein hydrolysates and their amino acid composition and biological activities in various fish are reviewed below.

## 3. Fish Muscle Protein Hydrolysates 

Fish protein hydrolysates are produced by chemical hydrolysis with either alkali or acid [24,25], fermentation with proteolytic microorganisms [26,27], or by digestion with enzymes [28,29,30,31]. 

Alkaline or acid hydrolysis results in reduced consumer acceptance due to the high variability of hydrolysates because the chemical cleavage of peptide bonds is not specific. Hydrolysis damages amino acid profiles by oxidizing cysteine and methionine, destroying some serine and threonine residues, and possibly converting asparagine and glutamine to aspartate and glutamate [32]. 

Fermentation with proteolytic microorganisms can enhance the nutritive and bioactive characteristics of fish matrices and improve human health benefits via the enzymatic activity of the raw material and the metabolic activity of microorganisms [27]. For instance, the beneficial aspects of consuming fermented fish protein products include antioxidant [33], ACE-inhibitory [34], anticancer [35], and antibacterial properties [33]. Ealier et al. found that fermentation causes a gradual decrease in the content of salt-soluble and water-soluble proteins in carp sausage, while increasing the content of free amino acids and insoluble proteins [36]. The advantage of fermentation is that microbial proteases hydrolyze the protein into bioactive peptides that can be purified without further hydrolysis. However, fermentation is limited due to low peptide yields. 

Enzymatic hydrolysis generates specific peptides by cleaving site-specific chains according to the specificity of the enzyme; this allows more efficient control of the manufacturing process by easily inactivating the enzyme after a specific degree of hydrolysis (DH) has been achieved [37]. The composition and subsequent physiological activities of the resulting hydrolysates are also highly influenced by the protein source, method and conditions of hydrolysis, and DH [38,39]. Thus, enzymatic hydrolysis is the most popular method for producing bioactive protein hydrolysates. Proteolytic enzymes are generally derived from plants, animals, and microbes. These include Alcalase [20], Neutrase [40,41], Protamex [42], Flavourzyme [43,44], bromelain [40,45], papain [40], pepsin [46], and trypsin [47]. Squid [48] and flounder [49] protein hydrolysates have been produced using esterase and Kojizyme. The functional properties of protein hydrolysates have been improved using gut extracts from catfish, squid, sleek hound, gray trigger, golden mullet, and goby.

Table 1 shows the various fish muscle protein hydrolysates used in proteolysis. Several protein hydrolysates are currently being produced from muscle proteins of monkfish ([47]), red lionfish (*Pterois volitans* L., [20]), cobia (*Rachycentron canadum*, [42]), catfish (*Pangasius hypophthalmus*, [40]), leatherjacket fish (*Meuchenia* sp., [45]), skipjack tuna (*Katsuwonus pelamis*, [44]), bighead croaker (*Collichthys niveatus*, [41]), Pacific whiting (*Merluccius productus*, [50]), ornate threadfin bream (*Nemipterus hexodon*, [51]), brownstripe red snapper (*Lutjanus vitta*, [43]), cuttlefish (*Sepia officinalis*, [52]), salmon (*Salmon salar*, [53]), sardinelle (*Sardina pilchardus*, [54]), thornback ray fish (*Raja clavate*, [55]), Pipefish (*Syngnathus schlegeli*, [46]), and flounder (*Daralichthys olivaceus*, [49]).

The antioxidant properties of protein hydrolysates of monkfish muscle have been evaluated [47]. Endogenous enzymes accelerate the hydrolysis of red lionfish muscle with 8.35% ± 0.96% DH using Alcalase and have generated 30.78% ± 1.57% DH within 30 min [20]. A comparison of cobia flesh hydrolyzed by Alcalase, Flavourzyme, and Protamex found that hydrolysates generated by Protamex have a higher DH and significant amounts of free tyrosine [42]. Protein hydrolysates have been generated from catfish or leatherjacket fish using bromelain, an enzyme derived from pineapple stems [40,45]. Liu et al. (2015) enzymatically hydrolyzed skipjack tuna muscle proteins with DH 2.43%, 33.80%, 56.72%, 71.68%, and 78.33% under controlled conditions using Alcalase, Flavorase, Neutrase, trypsin, and Protamex, respectively [44] and generated highly soluble fish protein hydrolysates comprising >80% protein. Shen et al. (2012) hydrolyzed bighead croaker muscle protein using Alcalase or Neutrase under specific temperature, pH, and enzyme-to-substrate ratios following degrees of hydrolysis and determined levels of sweet and umami amino acids produced from the hydrolysates of muscle protein in a study of response surface methodology [41]. Pacheco et al. (2008) generated muscle hydrolysates from Pacific whiting with 10%, 15%, and 20% DH using the commercial protease Alcalase at pH 4.0, 7.0, and 10 and found that peptide solubility increased proportionally with DH, whereas emulsifying properties and foaming capacity were not affected [50]. However, emulsion stability has been associated with DH [51,56,57]. Nalinanon et al. (2011) generated protein hydrolysates with 10%, 20%, and 30% DH from ornate threadfin bream muscle using skipjack tuna pepsin [51]. The authors emphasized that the emulsifying and foaming properties of the hydrolysates were controlled by their DH and the concentrations applied. Khantaphant, Benjakul, and Kishimura (2011) generated protein hydrolysates with 40% DH from brownstripe red snapper muscle using Alcalase or Flavourzyme as a first step, followed by hydrolysis with pyloric caeca protease (isolated from brownstripe red snapper muscle) as the second step [43]. Enzyme extracts from the digestive tract, namely the stomach, pyloric caeca, and intestine, have also been applied to prepare protein hydrolysates from fish muscles and characterize their functional properties [51,52,53]. Balti (2015) and Darewicz et al. (2014) have produced protein hydrolysates from cuttlefish [52] or salmon [53] using endogenous cuttlefish hepatopancreatic enzymes or human and porcine gastrointestinal enzymes. Jemil (2017) and Lassoued (2016) investigated protein hydrolysates from sardinelles and thornback ray fish (*Raja clavate*) generated by >7 *Bacillus subtilis* A26 proteases [54,55]. This strategy enhanced amounts of protein hydrolysates with abundant low-molecular-weight peptides. Wijesekara et al. (2011) and Ko et al. (2016) described muscle hydrolysates from pipefish and flounder generated by commercial proteolytic enzymes papain, Alcalase, Neutrase, Pronase, pepsin, trypsin, and Kojizyme and characterized their ACE-inhibitory activities [46,49]. 

## 4. Amino Acids in Fish Protein Hydrolysates

Proteolytic enzymes break down proteins into hydrolysates that comprise small peptides consisting of 2–20 amino acids [57]. The molecular weight, length, and sequence of the peptides and their amino acid composition influence their bioactive properties; hydrolysates produce the forms of amino acids that are useful in supporting various human biological functions [58,59]. Protein hydrolysates comprise free amino acids and short-chain peptides, which confer many benefits as functional foods due to their amino acid profiles. The amino acid composition of food proteins plays an important role in various human physiological activities and directly or indirectly affects the maintenance of human health. Amino acids are essential for the synthesis of various proteins with important functions, including oxygen carriers, vitamins, CO_2_, enzymes, and structural proteins [60,61]. 

The amino acid profiles of fish proteins play significant roles in various biological and physiological activities and in maintaining the health of humans. Amino acids such as aspartic acid, glycine, and glutamic acid enhance wound healing [62]. Tyrosine, methionine, histidine, lysine, and tryptophan have powerful radical scavenging activity in oxidative reactions [63]. Hydrophobic amino acids can act on membrane lipid bilayers to reach targets and help to scavenge radicals. Histidine significantly enhances antioxidant capacity because the protonation of the imidazole ring acts as a hydrogen donor [64]. Amino acids with antioxidant properties can chelate Fe^2+^ and Cu^2+^, thereby reducing their activity, and can inhibit lipid peroxidation [63]. The aromatic amino acids phenylalanine, tryptophan, and tyrosine possess considerable antioxidant activity and powerful chelating effects [65]. In the oxidative state, phenylalanine and tyrosine with phenyl and 4-hydroxyphenyl groups, respectively, and tryptophan with a heterocyclic indole ring stabilize the polypeptide structure through the π-stacking effect, act in the acid–base reaction as a part of a catalytic triad, and are involved in charge stabilization and electron relays. Glutamic and aspartic acids have potential antiproliferative activity against tumor cells [66], and low concentrations of aromatic amino acids rich in glycine and proline have more potent 2,2-diphenyl-1-picrylhydrazyl (DPPH) radical scavenging activity, possibly due to nonaromatic amino acids, such as glycine and proline [67]. Aspartic acid, glutamine, proline, glycine, and leucine contribute to powerful cytotoxicity towards cancer cells [63]. 

Fish proteins are easily digested and abundant in essential amino acids that are limited in terrestrial meat proteins such as methionine and lysine (6.5% vs. 5.7% and 19.6% vs. 19.0% of total essential amino acids in fish vs. terrestrial meat, respectively) [1]. Zou et al. (2016) tracked preferential amino acids for antioxidant activities among selected antioxidant peptides [63] and found that 33.7% of total amino acids were glycine, proline, and leucine; 18.7% were alanine, tyrosine, and valine; and 4.9% were methionine, glutamine, and cysteine. They emphasized that a high proportion of hydrophobic amino acids confers the ability of peptides to scavenge radicals. 

## 5. Amino Acid Composition of Fish Muscle Protein Hydrolysates

Table 2 shows the amino acid composition of hydrolysates generated from various fish muscle proteins. The amino acid composition of muscle hydrolysates has been determined in tuna dark muscle [68], red lionfish [20], capelin [69], Nile tilapia [70], Argentine croaker [21], black tilapia [71], round scad (Decanters maruadsi, [72]), yellow stripe trevally (*Selaroides leptolepis*, [73]), bighead croaker [41], whitemouth croaker [61], Atlantic salmon [74], coho salmon [74], and Alaska pollock [74].

Saidi et al. (2014) reported that among the hydrolysates from tuna dark muscle, the 1–4 kDa fractions with the strongest superoxide scavenging and reducing activities accounted for the high amounts of hydrophobic tyrosine, phenylalanine, proline, alanine, histidine, and leucine [68], corresponding to the results of Zou et al. (2016) [63]. The <1 kDa fraction of hydrolysate from red lionfish muscle, which has inhibitory activity against oxidative discoloration of β-carotene, contains abundant aromatic amino acids. Moreover, the 5–3 kDa fraction that contained a higher concentration of hydrophobic amino acids than other fractions had significant ACE-inhibitory capacity [20]. The amino acid profiles of capelin muscle hydrolysates generated by Alcalase and the original capelin are similar, except for methionine and tryptophan [69]. The amino acid profiles of hydrolysates generated from capelin (*Mallotus villosus*) myofibrils using Flavourzime and the original protein were similar [70]. Another study by Rocha et al. (2018) found that the hydrolysate obtained from Argentine croaker muscle by Alcalase exhibits the highest ABTS scavenging activity with a high content of hydrophobic amino acids (365.44 mg/g) compared to the hydrolysate obtained using Protamex [21]. They reported that hydrophobic amino acids in the hydrolysate inhibit free radicals as proton donors. In addition, the authors also explained that the increased ratios of aromatic amino acids in the hydrolysate can stabilize reactive oxygen species by transferring electrons and maintaining their stability through resonance to enhance antioxidant power based on the reduction of ferrous ions. The amino acids in hydrolysates of black tilapia muscle generated by Alcalase compared to those in the nonhydrolyzed fish protein displayed comparable protein quality for human nutrition, as suggested by the World Health Organization and Food and Agricultural Organization (1973) [71]. Thiansilakul et al. (2007) found that the high content of hydrophobic amino acids in round scad muscle hydrolysates could be a dietary protein supplement and might contribute to the bitterness of the hydrolysate [72]. The bitterness of hydrolysates containing abundant hydrophobic amino acids indicates that the composition of protein hydrolysates from yellow stripe trevally depends on enzyme specificity (Alcalase and Flavourzyme), and large amounts of proline increase bitterness in the hydrolysates compared with flesh. Glutamic acid, glycine, and aspartic acid might be responsible for the flavor and taste of amino acids in marine fish. Glutamic acid (10.85%), alanine (5.21%), and glycine (2.01%) probably contribute to the taste of hydrolysates from bighead croaker muscle and shellfish [41,75]. Abundant hydrophobic amino acids in whitemouth croaker (*Micropogonias furnieri*) hydrolysates increase its solubility in lipids, resulting in stronger antioxidant activity [61]. Nakajima et al. (2009) found ACE-inhibitory activity and slightly more hydrophobic amino acids in Atlantic salmon, coho salmon, Alaska pollock, southern blue whiting, and their hydrolysates generated by pepsin + pancreatin compared with pepsin [74]. 

## 6. Conclusions

The present tendency towards health consciousness among fish consumers has resulted in concern about the nutritional quality of fish, which is associated with the muscle protein content and their amino acids. Protein from fish muscle is easily digested and abundant in several essential amino acids compared with that from most terrestrial meats. The hydrolysate and amino acid profiles of fish muscle protein significantly impact numerous human biological and physiological activities. The composition and physical activities of the resulting hydrolysates are affected by the protein source (fish species), proteolytic enzymes, hydrolytic methods and conditions, and the DH. Amino acids in hydrolysates have various functional and bioactive properties.

## Figures and Tables

**Table 1:** Proteases used to produce fish muscle protein hydrolysates.

Source	Ref.	Protease	Enzymolysis Condition	DH	Functional and Biological Properties
Monkfish	[47]	Trypsin	Enzymolysis time 4 h, E/P 2%, Temp. 40 °C and pH 8.0	19.83% ± 0.82%	Hydroxyl radical scavenger
Red lionfish	[20]	Alcalase	Enzymolysis time 30, 60, and 90 min, E/P 0.3 AU/g protein, Temp. 50 °C and pH 8	30.78% ± 1.57%, 27.14% ± 1.20%, and 30.08% ± 0.25% (30, 60, and 90 min)	Antioxidant, chelator, angiotensin-converting enzyme inhibitor
Cobia (*Rachycentron canadum*)	[42]	Alcalase, Flavorase, Protamex	Alcalase (pH 8.0, 50 °C, E/P 99.75 U/g), Flavourzyme (pH 7.0, 50 °C, E/P 2.07 U/g) and Protamex (pH 7.0, 40 °C, E/P 8.41 U/g), Enzymolysis time 560 min	12.5%, 11.7%, 33.1%	-
Catfish (*Pangasius hypothalamus*)	[40]	Neutrase, Papain, Bromelain	Neutrase (50 °C, pH 6.5), Papain (55 °C, pH 7.5), Bromelain (55 °C, pH 6.5), Enzymolysis time 180 min	13.30%, 31.16%, 29.36%	-
Leatherjacket fish (*Meuchenia* sp.)	[45]	Bromelain, Papain, Flavourzyme	Bromelain, Papain (0.5% for water soluble protein and 1.5% for insoluble protein), Flavourzyme (0.25 % for water-soluble protein and 0.75% for insoluble protein), 50 °C, without any pH adjustment, 2 to 10 h	-	ACE inhibitor
Skipjack tuna (*Katsuwonus pelamis*)	[44]	Alcalase, Protamex, Flavorase, Neutrase	Alcalase (55 °C, pH 8), Protamex (50 °C, pH 7), Flavorase (55 °C, pH 7), Neutrase (50 °C, pH 7), Enzymolysis time 5 h, solid/liquid ratio 1:2	2.43%, 78.33%, 33.80%, and 56.72%	Solubilizer, emulsifier
Bighead croaker (*Collichthys niveatus*)	[41]	Alcalase, Neutrase	Alcalase (pH 8.0, 60 °C), Neutrase (pH 7.0, 50 °C), Enzymolysis time 4 h	17.03%, 15.04%	-
Pacific whiting (*Merluccius productus*)	[50]	Alcalase	pH 8.0, 50 °C, up to 2 h	10%, 15% and 20% (pH 4.0, 7.0 and 10, respectively)	Solubilizer, emulsifier, foaming capacity, foam stability
Ornate threadfin bream (*Nemipterus hexodon*)	[51]	Pepsin from skipjack tuna	pH 8.0, 50 °C, E/P 0.11 to 0.52 %, 1 h of hydrolysis	10%, 20% and 30% by adding skipjack tuna pepsin (140–650 μL)	ABTS and DPPH radical scavenger, chelator
Brownstripe red snapper (*Lutjanus vitta*)	[43]	Alcalase or Flavourzyme, as the first step and Pyloric caeca protease (PCP)	Alcalase (50 °C, pH 8.0), Flavourzyme (50 °C, pH 7.0), PCP (60 °C, pH 8.5), Enzymolysis time 10 min	40%	Antioxidant and ACE inhibitor
Cuttle fish (*Sepia officinalis*)	[52]	A21 proteases, Cuttlefish proteases	A21 proteases (pH 10.0; 50 °C), Cuttlefish hepatopancreas (pH 8.0; 50 °C), E/P 3:1 U/mg	16%, 8%	ACE inhibitor
Salmon (*Salmon salar*)	[53]	Human and Porcine gastrointestinal enzymes	-	-	ACE inhibitor
Sardinelle (*Sardina pilchardus*)	[54]	A26 proteases	pH 8.0; 45 °C, E/P 3:1 U/mg, Enzymolysis time 300 min	10%	Antibacterial, antioxidant and antihypertensive
Thornback ray fish (*Raja clavate*)	[55]	A26 proteases	pH 8.0; 40 °C, E/P 3:1 U/mg, Enzymolysis time 390 min	18%	ACE inhibitor
Pipefish (*Syngnathus schlegeli*)	[46]	Papain, Alcalase, Neutrase, Pronase, Pepsin and Trypsin	E/P 1%, Enzymolysis time 8 h	-	ACE inhibitor
Flounder (*Daralichthys olivaceus*)	[49]	Pepsin, Papain, Trypsin, Kojizyme	E/P 1:500	-	ACE inhibitor

E/P: enzyme/protein; ACE: angiotensin-converting enzyme; ABTS: 2,2′-azino-bis(3-ethylbenzthiazoline-6-sulfonic acid); DPPH: 2,2-diphenyl-1-picryl-hydrazyl-hydrate.

**Table 2:** Amino acid composition of fish muscle protein hydrolysates prepared from different fish species.

Amino Acid		Studies															
		Tuna Dark Muscle (g/100 g) [68]				Red Lionfish Muscle (g/100 g) [20]				Capelin % [69]				Nile Tilapia (g/kg) [70]			
Enzyme		Alcalase				Alcalase				Protein		Alcalase		Protein		Flavourzime	
Size		TPH		1–4 kDa		F 5–3 (3–5 kDa)		F < 1 (<1kDa)		-		-		-		-	
Protein content		72 ± 1.61 (g·L^−1^)		76 ± 1.59 (g·L^−1^)		-		-		13.9 ± 0.21		72.4 ± 0.70		823 g kg^−1^		899 g kg^−1^	
Aspartic acid		5.4 ± 0.1		7.5 ± 0.2		8.44 ± 0.80		8.80 ± 0.42		8.88 ± 0.15		9.89 ± 0.53		-		-	
Threonine		3 ± 0.1		4 ± 0.1		3.57 ± 0.02		4.58 ± 0.15		4.82 ± 0.05		4.56 ± 0.03		49		52	
Serine		2.3 ± 0.1		3 ± 0.32		2.97 ± 0.07		4.09 ± 0.12		4.18 ± 0.05		4.24 ± 0.10		-		-	
Glutamic acid		8.5 ± 0.2		12.1 ± 0.1		13.24 ± 0.54		11.00 ± 0.41		13.2 ± 0.03		13.4 ± 0.03		-		-	
Proline		2.2 ± 0.0		3 ± 0.2		14.52 ± 0.37		5.71 ± 0.57		3.70 ± 0.15		3.67 ± 0.03		-		-	
Glycine		3.6 ± 0.		4.8 ± 0.1		4.24 ± 0.19		4.48 ± 0.16		5.32 ± 0.04		5.14 ± 0.01		-		-	
Alanine		4.6 ± 0.2		6.1 ± 0.3		2.43 ± 0.04		2.21 ± 0.19		5.57 ± 0.04		6.00 ± 0.01		-		-	
Valine		3.2 ± 0.1		3.9 ± 0.1		14.86 ± 0.20		13.27 ± 0.02		5.71 ± 0.12		5.77 ± 0.01		51		53	
Methionine		1.7 ± 0.1		2.2 ± 0.2		-		3.12 ± 0.34		3.09 ± 0.02		2.05 ± 0.01		21		24	
Isoleucine		4.1 ± 0.6		4.0 ± 0.1		7.04 ± 0.22		8.68 ± 0.11		4.72 ± 0.08		4.25 ± 0.04		49		49	
Leucine		4.9 ± 0.2		6 ± 0.2		2.77 ± 0.11		2.64 ± 0.04		8.15 ± 0.05		7.60 ± 0.00		86		84	
Tyrosine		1.8 ± 0.1		2.2 ± 0.2		2.57 ± 0.05		3.42 ± 0.05		3.34 ± 0.01		2.47 ± 0.06		35		35	
Phenylalanine		2.4 ± 0.1		3 ± 0.2		3.11 ± 0.20		4.19 ± 0.09		3.80 ± 0.01		3.19 ± 0.00		43		44	
Histidine		2.4 ± 0.1		3 ± 0.2		1.23 ± 0.15		1.64 ± 0.12		2.43 ± 0.00		2.09 ± 0.02		24		23	
Lysine		4.7 ± 0.1		6.1 ± 0.1		7.74 ± 0.18		6.43 ± 0.09		8.47 ± 0.09		8.49 ± 0.06		105		92	
Arginine		2.4 ± 0.2		4 ± 0.21		11.13 ± 0.12		15.23 ± 0.40		5.99 ± 0.10		5.70 ± 0.02		-		-	
Tryptophan		0.3 ± 0.0		0.5 ± 0.0		-		0.51 ± 0.06		1.07 ± 0.01		0.43 ± 0.01		11		14	
Cystine		0.4 ± 0.1		0.4 ± 0.1		0.13 ± 0.18		-		1.33 ± 0.10		1.34 ± 0.00		6		8	
HAA		25.3		30.8		47.4		43.24		39.41		36.34					
AAA		4.5		5.7		5.68		8.12		8.21		6.09		89		93	
NCAA		13.9		19.6		21.68		19.8		22.08		23.29		-		-	
PCAA		9.5		13.1		20.1		23.3		16.89		16.28		-		-	
EAA		26.4		32.2		40.29		44.55		41.19		38		428		421	
	**Argentine Croaker** **(mg/g) [21]**				**Black Tilapia** **(mg/g) [71]**				**Round Scad (%) [72]**		**Yellow Stripe Trevally** **(%) [73]**						**Bighead Croaker** **(%) [41]**
**Enzyme**	**Alcalase**		**Protamex**		**AC**		**Alcalase**		**Flavourzyme**		**Protein**		**Alcalase**		**Flavourzyme**		**Neutrase**
Size	1083 Da		1350 Da		-		-		-		-		<7 kDa		7–8 kDa		-
Protein content	-		-		-		49.6 ± 0.19		69.0 ± 3.57		-		-		-		-
Aspartic acid	136.93 ± 0.38		136.73 ± 0.60		45.4 ± 0.16		67.2 ± 5.30		2.04		10.01		9.55		9.40		1.91
Threonine	45.38 ± 0.62		44.54 ± 0.15		20.1 ± 6.42		30.2 ± 6.89		5.09		2.92		5.35		5.40		10.92
Serine	46.04 ± 0.40		49.08 ± 0.41		21.5 ± 0.78		24.0 ± 0.10		8.16		5.46		5.21		5.15		3.36
Glutamic acid	188.60 ± 1.37		196.19 ±0.33		141 ± 6.48		151 ± 7.05		3.47		9.88		13.77		13.89		10.85
Proline	33.37 ± 0.46		31.71 ± 0.45		17.5 ± 2.42		22.6 ± 2.98		0.51		3.33		3.81		3.84		0.72
Glycine	35.87 ± 0.28		36.25 ± 0.12		31.8 ± 3.16		40.9 ± 1.66		1.49		16.48		8.87		8.65		2.01
Alanine	58.40 ± 0.04		62.31 ± 0.35		32.4 ± 0.99		37.7 ± 2.01		5.31		9.64		9.49		9.46		5.21
Valine	35.87 ± 0.02		34.02 ± 0.21		23.0 ± 0.25		31.9 ± 0.56		6.77		2.77		3.61		3.38		5.12
Methionine	41.68 ± 0.48		39.33 ± 0.21		19.9 ± 2.09		27.6 ± 3.01		4.51		1.76		2.58		1.87		4.19
Isoleucine	31.00 ± 0.09		26.24 ± 0.09		24.4 ± 3.15		33.6 ± 1.26		3.15		4.31		4.14		4.49		4.24
Leucine	83.96 ±0.19		81.28 ± 0.30		56.0 ± 0.78		68.9 ± 0.11		10.1		6.72		8.38		8.58		7.29
Tyrosine	38.32 ± 0.11		34.15 ± 0.09		16.4 ± 2.69		25.8 ± 1.08		5.20		5.62		5.70		6.11		9.86
Phenylalanine	38.67 ± 0.01		40.92 ± 0.22		52.0 ± 2.43		59.1 ± 2.81		4.52		3.08		2.61		2.66		10.79
Histidine	24.81 ± 1.38		24.69 ± 0.10		-		-		11.2		5.49		3.62		2.98		1.93
Lysine	96.07 ± 0.36		97.98 ± 0.05		50.3 ± 0.04		75.9 ± 1.11		13.9		8.45		8.35		8.72		9.76
Arginine	61.01 ± 0.46		59.62 ± 0.04		34.4 ± 2.42		49.1 ± 0.50		14.0		2.64		3.50		3.88		5.79
Tryptophan	-		-		-		-		-		-		-		-		3.55
Cystine	4.16 ± 0.01		4.96 ± 0.04		-		-		0.69		1.43		1.47		1.53		2.49
HAA	365.44 ± 0.34		354.92 ± 0.15		241.6		307.2		40.76		38.66		41.79		41.92		49.91
AAA	77.00 ± 0.11		75.07 ± 0.13		68.4		84.9		9.72		8.7		8.31		8.77		24.2
NCAA	325.53 ± 1.75		332.92 ± 0.92		186.4		218.2		5.51		19.89		23.32		23.29		12.76
PCAA	181.74 ± 0.68		182.29 ± 0.09		84.7		125		39.1		16.58		15.47		15.58		17.48
EAA	390.23 ± 0.28		389.00 ± 0.40		245.7		327.2		59.24		35.5		38.64		38.08		54.24
	**Whitemouth Croaker (mg/g) [61]**		**Atlantic Salmon** **(mg/100 g) [74]**				**Coho Salmon** **(mg/100 g) [74]**				**Alaska Pollock** **(mg/100 g) [74]**				**Southern Blue Whiting (mg/100 g) [74]**		
**Enzyme**	**Alcalase**		**Pepsin**		**Pepsin +** **pancreatin**		**Pepsin**		**Pepsin +** **pancreatin**		**Pepsin**		**Pepsin +** **pancreatin**		**Pepsin**		**Pepsin +** **pancreatin**
Size	2–34 kDa		-		-		-		-		-		-		-		-
Protein content	82.3 ± 0.8		^a^633–1020		^a^1610–2530		^a^633–1020		^a^1610–2530		^a^633–1020		^a^1610–2530		^a^633–1020		^a^1610–2530
Aspartic acid	105.23 ± 0.86		0.4 ± 0.2		11.6 ± 1.1		1.4 ± 0.2		17.8 ± 1.3		11.8 ± 0.7		16.9 ± 5.3		5.9 ± 1.2		1.1 ± 0.6
Threonine	48.58 ± 0.58		2.9 ± 0.2		6.0 ± 0.3		2.8 ± 0.1		7.3 ± 0.4		13.5 ± 0.2		24.2 ± 6.0		4.9 ± 0.4		2.8 ± 0.9
Serine	39.47 ± 1.63		4.8 ± 0.2		6.4 ± 0.3		6.1 ± 0.1		8.3 ± 0.4		18.3 ± 1.1		6.2 ± 1.4		7.6 ± 1.1		0.6 ± 0.4
Glutamic acid	173.11 ± 4.93		3.1 ± 0.1		29.0 ± 1.2		6.4 ± 0.2		45.9 ± 2.2		3.1 ± 0.1		29.0 ± 1.2		32.9 ± 5.5		5.8 ± 1.7
Proline	39.33 ± 0.94		-		-		-		-		-		-		-		-
Glycine	40.54 ± 0.19		13.7 ± 0.8		15.2 ± 1.0		28.3 ± 0.1		26.0 ± 3.6		36.7 ± 1.2		55.4 ± 11.6		19.1 ± 2.5		19.0 ± 3.7
Alanine	57.10 ± 1.49		24.7 ± 1.5		35.5 ± 0.9		26.7 ± 0.6		35.9 ± 2.8		37.2 ± 1.2		58.9 ± 13.5		36.9 ± 5.3		10.4 ± 1.9
Valine	52.52 ± 1.13		2.5 ± 0.1		15.2 ± 0.3		2.3 ± 0.2		20.1 ± 3.2		5.1 ± 0.2		20.0 ± 5.2		5.4 ± 0.8		10.3 ± 2.2
Methionine	39.63 ± 0.58		1.3 ± 0.1		11.8 ± 0.4		1.9 ± 0.1a		14.2 ± 0.6		5.8 ± 0.4		21.6 ± 4.9		5.5 ± 1.0		17.6 ± 3.3
Isoleucine	42.77 ± 2.48		1.2 ± 0.1		21.9 ± 2.2		1.1 ± 0.1		17.4 ± 4.7		3.4 ± 0.1		17.4 ± 3.9		4.4 ± 0.7		10.2 ± 1.8
Leucine	96.42 ± 2.75		2.4 ± 0.1		84.3 ± 1.4		2.7 ± 0.2		88.8 ± 2.2		7.0 ± 0.2		90.3 ± 23		6.7 ± 0.9		63.4 ± 13.3
Tyrosine	-		1.9 ± 0.1		76.9 ± 4.3		3.1 ± 0.1		67.4 ± 8.1		3.9 ± 0.1		62.8 ± 17		3.2 ± 0.5		50.0 ± 11.3
Phenylalanine	44.43 ± 0.33		3.1 ± 0.3		76.3 ± 5.3		2.4 ± 0.1		82.0 ± 5.1		3.8 ± 0.3		62.6 ± 16		3.7 ± 0.5		47.5 ± 11.4
Histidine	23.74 ±2.46		16.4 ± 0.9		21.0 ± 0.8		24.9 ± 0.1		27.8 ± 1.3		9.1 ± 0.4		11.9 ± 3.0		2.2 ± 0.4		3.1 ± 0.8
Lysine	97.85 ± 1.41		1.9 ± 0.2		130.0 ± 8.0		2.7 ± 0.2		141 ± 8.0		20.5 ± 0.8		86.5 ± 24		0.6 ± 0.1		45.9 ± 11
Arginine	59.36 ± 0.52		1.3 ± 0.6		196 ± 14		1.5 ± 0.2		204 ± 13		9.3 ± 0.3		215 ± 57		3.2 ± 0.4		132 ± 30
Tryptophan	34.81 ± 0.66		n.d.		18.3 ± 0.5		1.4 ± 0.1		19.4 ± 0.5		n.d.		n.d.		n.d.		n.d.
Cystine	5.09 ± 0.31		n.d.		1.7 ± 0.1		n.d		1.9 ± 0.1		n.d.		n.d.		n.d.		n.d.
HAA	377.29		37.1		323.6		40.2		327.7		66.2		333.6		65.8		209.4
AAA	79.24		5		171.5		6.9		168.8		7.7		125.4		6.9		97.5
NCAA	278.34		3.5		40.6		7.8		63.7		14.9		45.9		38.8		6.9
PCAA	180.94		19.6		347		29.1		372.8		38.9		313.4		6		181
EAA	445.88		31.7		366.5		40.8		398.6		68.2		334.5		33.4		200.8

TPH: total hydrolysate; 1–4 kDa: 1–4 kDa membrane peptide fractions; F 3–5: 3–5 kDa fractions; F <1: < 1 kDa fractions; AC: hydrolysate powders from control hydrolysis without enzyme Alcalase; HAA: hydrophobic amino acids (alanine, valine, isoleucine, leucine, tyrosine, phenylalanine, proline, methionine, and cysteine); AAA: aromatic amino acids (phenylalanine, tryptophan, and tyrosine); NCAA: negatively charged amino acids (aspartic acid, glutamic acid); PCAA: positively charged amino acids (arginine, histidine, lysine); EEA: essential amino acids (phenylalanine, valine, threonine, isoleucine, methionine, histidine, leucine, and lysine. ^a^ Extractive nitrogen content.
